# Knockdown of *NeuroD2* leads to seizure-like behavior, brain neuronal hyperactivity and a leaky blood-brain barrier in a *Xenopus laevis* tadpole model of DEE72

**DOI:** 10.1093/genetics/iyae085

**Published:** 2024-05-24

**Authors:** Sulagna Banerjee, Paul Szyszka, Caroline W Beck

**Affiliations:** Department of Zoology, University of Otago, PO Box56, Dunedin 9016, New Zealand; Department of Zoology, University of Otago, PO Box56, Dunedin 9016, New Zealand; Brain Health Research Centre, University of Otago, Dunedin 9016, New Zealand; Department of Zoology, University of Otago, PO Box56, Dunedin 9016, New Zealand; Brain Health Research Centre, University of Otago, Dunedin 9016, New Zealand; Genetics Otago Research Centre, University of Otago, Dunedin 9016, New Zealand

**Keywords:** epilepsy, NeuroD2, blood-brain barrier, Losartan, developmental and epileptic encephalopathy, C-starts, Mauthner neuron, C-shaped contractions, C-SC

## Abstract

Developmental and Epileptic Encephalopathies (DEE) are a genetically diverse group of severe, early onset seizure disorders. DEE are normally identified clinically in the first six months of life by the presence of frequent, difficult to control seizures and accompanying stalling or regression of development. DEE72 results from de novo mutations of the *NEUROD2* gene that result in loss of activity of the encoded transcription factor, and the seizure phenotype was shown to be recapitulated in *Xenopus tropicalis* tadpoles. We used CRISPR/Cas9 to make a DEE72 model in *Xenopus laevis*, to further investigate the developmental etiology. *NeuroD2.S* CRISPR/Cas9 edited tadpoles were more active, swam faster on average, and had more seizures (C-shaped contractions resembling unprovoked C-start escape responses) than their sibling controls. Live imaging of Ca^2+^ signaling revealed prolongued, strong signals sweeping through the brain, indicative of neuronal hyperactivity. While the resulting tadpole brain appeared grossly normal, the blood-brain barrier (BBB) was found to be leakier than that of controls. Additionally, the TGFβ antagonist Losartan was shown to have a short-term protective effect, reducing neuronal hyperactivity and reducing permeability of the BBB. Treatment of *NeuroD2* CRISPant tadpoles with 5 mM Losartan decreased seizure events by more than 4-fold compared to the baseline. Our results support a model of DEE72 resulting from reduced NeuroD2 activity during vertebrate brain development, and indicate that a leaky BBB contributes to epileptogenesis.

## Introduction

Epilepsy is one of the most common neurological disorders, estimated to affect 1% of the population at some time in their lives. Seizures, the defining feature of epilepsy, are visible manifestations of the underlying hyper-excitability of neuronal firing in the brain (Gross 1992). Patients are generally treated with one or more antiseizure drugs (ASD) which aim to reduce or eliminate seizures. However, in many patients, seizure control cannot be achieved, and the ongoing and unpredictable nature of seizure occurrence can result in poor quality of life. The developmental and epileptic encephalopathies (DEE), which present in infancy or very early childhood, are the most severe group of genetic epilepsies (McTague *et al*. 2016). DEE incorporates previously described syndromes of infantile onset epilepsy such as Otahara, Dravet, and West syndromes, as well as later onset syndromes such as Lennox Gastaut (reviewed in Scheffer and Liao (2020)). Diagnosis is confirmed by the presence of seizures, abnormal electroencephalogram (EEG) activity that persists between seizures, and stalling, delay of regression of cognitive and behavioral development (Scheffer *et al*. 2017; Scheffer and Liao 2020). Mortality is 25% by age 20 (Camfield and Camfield 2008), and survivors often have significant lifelong intellectual and motor disabilities, requiring lifelong care (Keezer *et al*. 2016).

DEE are all linked by a similar clinical presentation: early onset, unrelenting seizures that are refractory to treatment. Individual DEE are ultra-rare, but the estimated cumulative incidence of DEE is 169 in 100,000 (Poke *et al*. 2023). Genome and whole-exome sequencing of patients with early onset epilepsy (e.g. Epi4K *et al*. 2013; Epi 2016), has identified 110 genes with rare variants that cause DEE, curated in Online Mendelian Inheritance in Man database (OMIM). However, this may just be the tip of the iceberg, as a recent review manually curated 936 genes associated with monogenic epilepsy, implicating 4% of human protein coding genes (Oliver *et al*. 2023). Of these, 818 (89%) were DEE-associated, with around 30% autosomal dominant inheritance (likely de novo) 60% autosomal recessive with the remaining 10% X-linked or mitochondrial (Oliver *et al*. 2023).

Functional studies in animal or cell models are lacking for the majority of DEE, and with so many genes and variants implicated, new approaches are needed to manage these severe conditions. Recent work demonstrates that the tadpole is a good model for human brain development (Willsey *et al*. 2021; Ta *et al*. 2022) as vertebrate central nervous system (CNS) development follows remarkably conserved mechanisms (reviewed in Exner and Willsey (2021)). In fact, much of what we know of vertebrate CNS development was discovered from studies in *Xenopus* and other amphibian embryos. The large size, plentiful number and external development of *Xenopus* eggs makes them amenable to genetic manipulation and has led to accurate fate maps allowing this to be targeted. Furthermore, early functional studies in *Xenopus* led to the current understanding of neural induction, patterning and neurogenesis in vertebrates (reviewed in Exner and Willsey (2021)). The week-old *Xenopus* tadpole brain is organized similarly to the human brain, with the main difference being the expansion of forebrain into the six-layered neocortex of humans, which is formed from the inside out via a process of radial migration, and generating the sulci and gyri (Molnar *et al*. 2006; Paridaen and Huttner 2014). *Xenopus* forebrains are lissencephalic (smooth), unlayered, and form from the outside in, so the oldest neurons are found on the outside (Moreno and Gonzalez 2017). While higher cognitive behaviors cannot be studied in *Xenopus*, the tractability of the tadpole system, combined with direct access to the brain at all developmental stages, make it a powerful model system for investigating the origins of human disorders arising from aberrant brain development (Pratt and Khakhalin 2013). Recent examples where *Xenopus* tadpoles have been used as models of human brain disorders include autism (Willsey *et al*. 2020, 2021), fragile X syndrome (Truszkowski *et al*. 2016), and DEE (Yoo *et al*. 2017; Sega *et al*. 2019).

Here, we set out to build on previous work by Sega *et al.* (2019), who used *X. tropicalis* tadpoles to show that two de novo pathogenic variants in *NEUROD2* cause DEE72. *Xenopus laevis* tadpoles have a well-developed brain at Nieuwkoop and Faber (NF) stage 47, which is about one week of age, and prefeeding. Seizures can be chemically induced by adding pentyleltetrazole (PTZ) or 4-aminopyrolidine (4-AP) to the swimming medium (Hewapathirane *et al*. 2008; Bell *et al*. 2011; Panthi *et al*. 2023). Additionally, since the skull has yet to form at this stage, the brain is easily accessible for electrode recording of local field potentials or Ca^2+^ signaling (Hewapathirane *et al*. 2008). The tadpoles can be housed individually in wells of a 24-well culture plate with 1 ml of medium, for automated behavioral classification and drug testing (Panthi *et al*. 2023). We wanted to see whether automated quantification of tadpole behavior would be sensitive enough to enable drug testing for antiseizure activity in this model. We also showed that brain hyperactivity, characteristic of spontaneous seizures, can be detected in live tadpole brains using the genetically encoded Ca^2+^ sensor GCaMP6s. While brain morphology appeared unaltered in CRISPants, the blood-brain barrier (BBB) was less able to prevent the loss of sodium fluorescein (NaF) dye injected into the ventricle, suggesting that a leaky BBB may contribute to the epileptogenic brain of DEE72 children. Our findings show that *X. laevis* tadpoles are a useful model of DEE72, and may be useful in future to develop models of other DEE for functional study. These models could be used to inform and undertake preclinical testing of repurposed drugs, such as Losartan, which may have the potential to protect the brain from the damaging effects of unrelenting seizures, thereby improving the quality of life for patients.

## Materials and methods

### Production of *Xenopus laevis* eggs and embryos

Animal procedures were approved by the University of Otago's Animal Ethics Committee under animal use procedures AUP19/01 and AUP22/12. Adult female *Xenopus laevis* were induced to lay eggs by injecting with 500 IU per 75 g body weight of Human chorionic gonadotrophin (Chorulon) into the dorsal lymph sac, 16 hours before eggs were required. They were placed in a dark incubator overnight in pairs in small holding tanks containing “frog water” (tap water passed through carbon filters to remove chlorine). After egg laying commenced, each female was placed in 1 L of 1 x Marc's modified ringers (MMR: 10 mM NaCl, 0.2 mM KCl, 0.1 mM MgSO_4_.6H_2_0, 0.2 mM CaCl_2_, 0.5 mM HEPES, 10 µM EDTA, pH 7.8) and eggs collected hourly and fertilized using fresh testes from a euthanized adult *X. laevis* male. Jelly coats were removed immediately following embryo rotation (15–20 minutes), using a 2% solution of Cysteine HCl (pH 7.9) and the resulting de-jellied embryos rinsed three times in MMR. Embryos were raised in small batches in 0.1 x MMR with no antibiotics and staged according to Nieuwkoop and Faber’s (1967) staging series. Testing was carried out at stage 47, which has been used previously in the tadpole-induced seizure model (Hewapathirane *et al*. 2008; Panthi *et al*. 2023).

### CRISPR/Cas9 targeting of *NeuroD2*

ChopChop v2 (Labun *et al*. 2016) was used to design short guide RNA (sgRNA). As *X. laevis* is allotetraploid (Session *et al*. 2016; Fisher *et al.* 2023), the sgRNA was designed to the *S* form, which is more highly expressed in tadpole brains (Ta *et al.* 2022). SgRNA were chosen based on specificity and ability to edit both *L* and *S* homeologs (with no other chromosomal off-targets). sgRNAs rnk5 and rnk20 (ranked 5th and 20th, respectively, for efficiency by ChopChop) met these criteria and were also located near the DEE72 human variant sites. Editing efficiency was predicted using InDelphi (Shen *et al.* 2018) (https://indelphi.giffordlab.mit.edu/), (Supplementary Fig. 1 in Supplementary File 2) which is a good editing predictor for *Xenopus* (Naert *et al.* 2020). *NeuroD2* sequences for human and *X. laevis* L and S proteins and *X. laevis NeuroD2*.*S* mRNA were obtained from NCBI and imported into SnapGene v5.2.4. Protein sequences were aligned in SnapGene using MUSCLE alignments and the bHLH, NLS domains and human DEE-causing variants annotated from the human sequence and data in previously published studies (Sega *et al.* 2019; Mis *et al.* 2021). Using the InDelphi (Shen et al. 2018) online prediction tool, 60 bp up- and downstream of the target Cas9 site in *X. laevis NeuroD2*.S were entered in “single mode” using mESC cell type, for each sgRNA. The EnGen sgRNA template oligo designer tool (NEB) was used to design long 54–55 bp oligonucleotides for sgRNA synthesis, with the 20-nucleotide sequence from ChopChop v.2 (without the “NGG” PAM). A “G” is added to the 5′ end of the target sequence if not present, as this enables optimal transcription. Primer and sgRNA oligonucleotide sequences can be found in Table 1, predicted edits for each sgRNA can be found in Supplementary Fig. 1 in Supplementary File 2.

**Table 1.** sgRNA sequences and amplification/sequencing primers for this study.

sgRNA rnk ChopChop v2	sgRNA sequence (PAM)	Forward primer	Reverse primer	Amplicon size
Rnk5	GGCGAGAGGCCCAAAAAACGTGG	ATACAGAAGGGTCTCTGGGTGA	TATCTTGGACAGCTTCTGGGTT	287bp
Rnk20	CTGAGTTCAGGTCGTGCATGCGG	ATACAGAAGGGTCTCTGGGTGA	ACAGCTTCTGGGTTTTGGAGTA	279bp

Oligonucleotides were ordered from Integrated DNA Technologies (IDT), and used with the EnGen sgRNA synthesis kit, *S. pyogenes* (NEB) to make sgRNA. The resulting sgRNA was dissolved in water, stored as small aliquots at −80°C and thawed on ice immediately prior to use. 0.3 µl of EnGen *S.pyogenes* Cas9 NLS enzyme (protein) was added to sgRNA, resulting in a 3- to 5-fold dilution, and incubated at 37°C for 5 minutes. The resulting mixture was loaded by back-filling into a pulled Drummond glass capillary needle. Fertilized, de-jellied embryos within 1 hour of fertilization were checked for sperm entry points and lined up in a row in a 2 mm × 40 mm trench cut into a 50 mm petri dish lined with 2.4 ml of 2% agar and filled with 6% Ficoll PM400 in MMR and placed under a dissecting microscope for microinjection. 9.2 nl of Cas9/sgRNA were injected into each egg near, but not into, the female pronucleus, using a Drummond Nanoject II injector held with a Narishige MM3 micromanipulator. Total amount of sgRNA per egg was 400 pg for the rnk5 and 900 pg for the rnk20 sgRNA. Eggs were injected in batches of 25 and immediately placed into the incubator at 18°C following injection. Each concentration of sgRNA was injected into 100 embryos from the same sibship, and controls were generated using only Cas9 enzyme (no sgRNA). After 2–3 hours, the embryos were checked for normal development and moved to 3% Ficoll in 0.1 x MMR. The following day embryos were checked for normal development, and five embryos were chosen at random for genotyping. The remainder were moved to 0.1 x MMR until stage 46–47 was reached. Genotyping was done by homogenizing each embryo or tadpole in 200 µl 5% Chelex beads in phosphate-buffered saline with 1.5 µl Proteinase K (25 mg/ml) and incubating for 3 hours (embryos) to overnight (tadpole) at 65°C. The reaction was then terminated by incubating samples at 95°C for 5–10 minutes, and briefly centrifuged to pellet the beads. 1 µl of the supernatant was used directly for PCR (for primers, see Table 1). TIDE analysis (Brinkman et al. 2014) (https://tide.nki.nl/) of CRISPant samples vs controls, which only received Cas9 protein, was used to confirm editing and InDelphi predictions.

### Recording and analysis of stage 47 tadpole seizure behavior

**Fig. 1.**
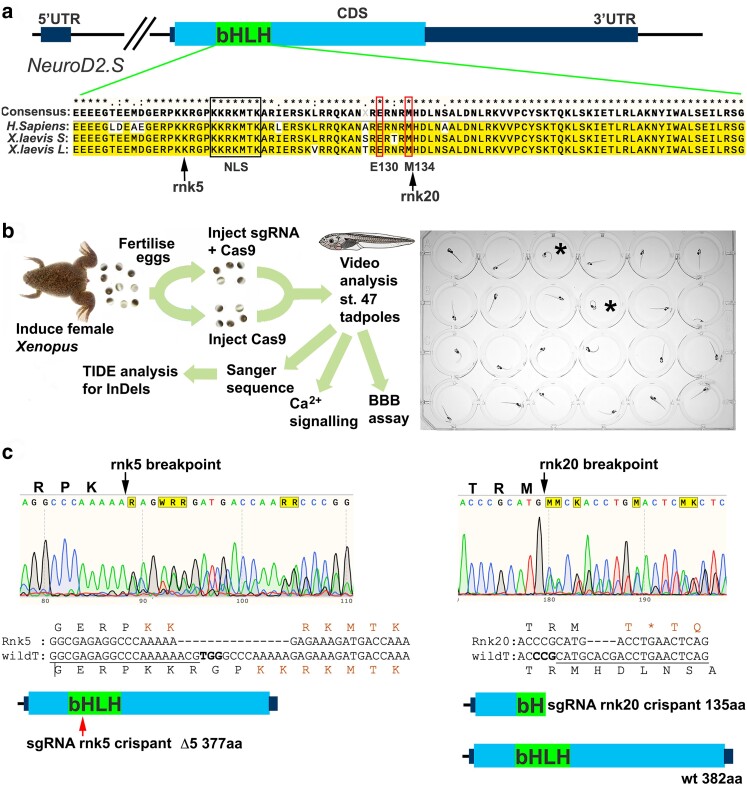
CRISPR/Cas9 editing of NeuroD2 in tadpoles. a) Schematic of the *X. laevis NeuroD2.S* gene, the homeolog *NeuroD2.L* is the same for all key aspects shown, including sgRNA binding sites. The NeuroD2 transcription factor is encoded by a single exon (CDS) and the bHLH DNA binding domain is indicated. Alignment of the bHLH domain between *H. sapiens* and *X. laevis* shows a high degree of conservation at protein level. Nuclear localization sequence (NLS) is indicated by a black box, and the conserved positions of the previously described de novo human variants E130Q and M134T that cause DEE72 (Sega *et al.* 2019) indicated by thin red boxes. The approximate sites of Cas9 cleavage sites of the sgRNA used in this study are indicated by arrows. b) Schematic of the experimental process. C-SC are indicated in two tadpoles (black asterisk). c) Example Sanger sequence traces with Cas9 breakpoints for both sgRNA indicated by black arrows. The most common outcomes for each guide are shown as sequence and protein schematics: rnk5 deletes 15 bp leading to a loss of 5 amino acids in the bHLH DNA binding domain, indicated by the red arrow. The NLS KKRKMTK is reformed around this deletion. Rnk20 sgRNA causes a 4 bp deletion which results in a premature STOP codon (orange asterisk) and a truncated protein lacking much of the DNA binding domain is predicted.

**Fig. 2.**
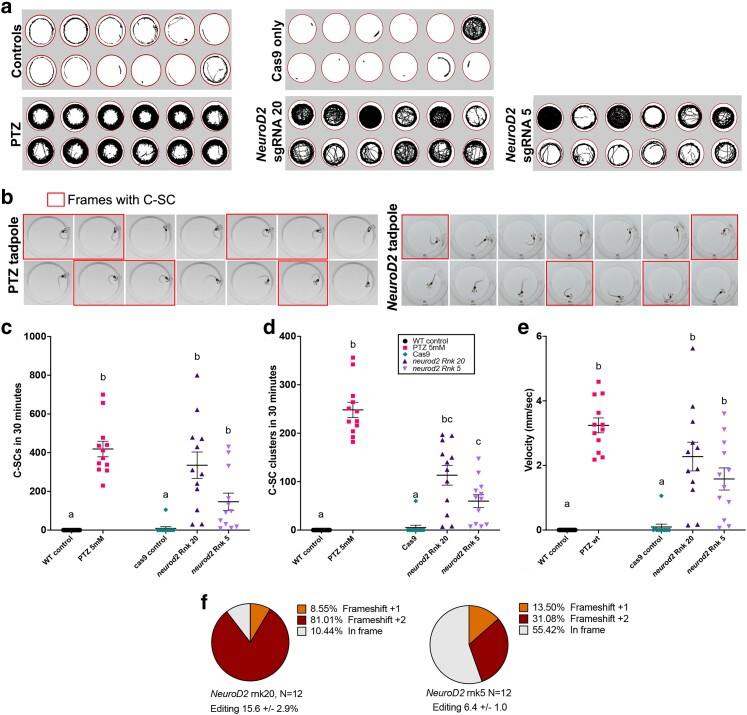
CRISPR/Cas9 editing of *NeuroD2* in tadpoles results in increased activity and C-SC of the tail. a) Tracks showing swimming trajectories of 12 individual tadpoles in wells of a 24-well plate (indicated by red circles), recorded over 30 minutes. b) 14 consecutive video frames from example tadpoles, each frame is 40 msec apart. Frames where the tadpole is undergoing a C-SC of the tail are outlined in red. Left: wild type tadpole treated with 5 mM PTZ to evoke acute C-SC; right: *NeuroD2* CRISPant tadpole with spontaneous C-SC. c–e) Scatter plots of behavioral data from groups of 12 tadpoles, analysed from 30 minutes of video data at 25 frames per second. Kruskal–Wallis statistical analysis with Dunn's post hoc testing of all means. Treatment groups that share the same lower-case letter are not significantly different, legend in d applies to all graphs. c) Manually counted C-SC frames from 30 minute video recordings, d) Manually counted clusters of C-SC seizures e) Mean velocity (mm/sec). f) Summary of editing types detected by TIDE in the *NeuroD2* CRISPant tadpoles used to generate these data. Raw count data and statistical analyses can be found in Supplementary File 1.

Individual tadpoles were transferred to a 24-well culture plate with each well containing 1 ml of 0.1 x MMR, enough to submerge tadpoles, allowing free swimming. A Panasonic DMC-FZ1000 camera was mounted on a tripod above the tadpoles and the Movie mode was used to capture 30 minutes of MP4 video at 25 frames per second. Tadpoles were backlit with an array of 176 ultrabright LED lights (Neewer, 1,300 lumens). In TopScan, the locomotion super module was used to create 24 arenas, and a clear background. Swimming tracks were generated for each tadpole, representing 30 minutes of activity. Mean velocity (mm/sec) was calculated by TopScan for each tadpole as in Panthi et al. (2023). C-shaped contractions (C-SC) over a 10-minute period were determined by the software counting the total number of C-SC events that met both the elongation ratio (<1.5) and motion measure (>0.2) criteria, and confirmed by manual checks. Examples of postures scored as C-SC in video frames can be found in Figs. 1b and 2b, with a positive score if the tadpole's head and tail described more than half of a complete circle. C-SC were also grouped into clusters, where multiple C-SCs frames were detected with an interval of less than 2 seconds between them.

### Tadpole group assignment

For *NeuroD2* CRISPant tadpoles, following visual confirmation of seizure behavior in 10 cm petri dishes, tadpoles were arbitrarily selected for behavioral recording and/or assigned to treatment groups. Sequencing of the *NeuroD2* locus of each tadpole was used to confirm that there were no significant editing differences between groups. Wild type or Cas9 only injected controls were also selected arbitrarily, and were from the same sibship as CRISPants.

### Ca^2+^ imaging and quantification

GCaMP6 s and mCherry in CS2 + plasmid (Li et al. 2022) were a kind gift from Edward Ruthazer and Anne Schohl. Both were linearized with NotI HF restriction enzyme (NEB) and mRNA was synthesized with the Invitrogen Message Machine SP6 kit. A total of 500 pg GCaMP6 s and 250 pg mCherry mRNA was injected bilaterally into 1-cell stage *X. laevis* embryos. According to Li et al. (2022) the signals from mRNA injection of GCaMP6 s are clearly inducible and detectable for at least one week past stage 48. At stage 47, tadpoles were initially anesthetized by immersion in a petri dish containing a 1:4,000 dilution of MS222 for a period of 2 minutes. Once movement ceased, tadpoles were transferred to a clean petri dish and surplus liquid removed. Tadpoles were then embedded in 40 µl 2% low melting point (LMP) agarose and immobilized by submerging in 0.1 x MMR containing 100 µM of the neuromuscular blocker pancuronium bromide (Sigma). After 5–10 minutes, fine, sharpened jeweler's forceps (Dumont #5) were used to gently remove the skin above the brain, which was then covered with a thin layer of 1% LMP agarose. Prepared tadpoles were positioned beneath a fluorescence microscope (Axio Examiner D1; Carl Zeiss), and brains imaged through a 50x magnification lens (50x/0.55 DIC EC Epiplan-Neofluar, Zeiss). GCaMP6 s fluorescence was excited at 480 nm by a monochromator (Polychrome V; T.I.L.L. Photonics). The excitation light intensity was reduced to 10% of its maximum. The emitted light was detected with a CCD camera (Sensicam; PCO Imaging) through a 495 nm dichroic mirror and a 505 nm long-pass filter. Pixels were binned on chip (4 × 4), resulting in an image resolution of 344 × 260 pixels. Images were acquired at a frame rate of 2 frames per second with an exposure time of 400 msec per frame. Baseline brain activity was recorded for 30 minutes for both Cas9 control and *NeuroD2* CRISPant tadpoles. Cas9 control tadpoles were subsequently exposed to 15 mM PTZ to induce acute seizures as a positive control. All tadpoles remained viable at the conclusion of the experimental procedures.

Imaging data were analysed with FiJi software (Schindelin et al. 2012). Ca^2 +^ signals were calculated as relative fluorescent change for each frame i of the recording as ΔF/F = (F_i_−F_B_)/F_B_, where F_i_ is the absolute fluorescence of the i^th^ frame and F_B_ is the background fluorescence, which was calculated as the median fluorescence of the entire recording. Using standard region-of-interest (ROI) parameters, Ca^2+^ signals from the midbrain were calculated by averaging ΔF/F % for each frame. “Large” (>5 ΔF/F %) and “small” (1–5 ΔF/F %) spikes were annotated manually. Total spike counts (small plus large) were compared between groups using a t*-*test with Mann–Whitney corrections.

### BBB permeability assay

To assess BBB permeability, stage 47 tadpoles were anesthetized and embedded in LMP agarose, as for Ca^2+^ signaling, covering the brain with a thin layer. Once the agarose had set, embedded tadpoles were submerged in 0.1 x MMR and 9.2 nl of 0.1 mg/ml NaF was injected into the 4th ventricle using a capillary needle (as for embryo injections). Tadpoles were protected from light following injection. A Leica Fluo III dissecting microscope with GFP2 filter set was used to image the tadpole head from the dorsal side at 2, 5, 10, 20, and 30 minutes postinjection using DFC7000T camera with standardized settings. Images were analysed offline using FiJi. The mean fluorescent intensity (MFI) was calculated from each image using the green channel across a defined region of interest (ROI). The ROI was located to the left side of the tadpole brain, and captures NaF dispersion across the BBB. A 2-way repeated measure ANOVA with Tukey post hoc multiple comparisons tests of all means was used to compare the MFI, representing NaF dispersion, at 2 and 20 minutes post injection.

### Chemical treatments

#### Acute seizure induction with pentylenetetrazole

Healthy, normally developing stage 47 tadpoles were arbitrarily selected and placed in 0.5 ml 0.1 x MMR in separate wells of a 24-well plate. To induce acute seizures/status epilepticus as described in (Hewapathirane et al. 2008), the seizure-inducing drug PTZ was made up in Milli Q water (MQW) at 100 and 25 µl added to individual tadpole wells to give a final concentration of 5 mM. This dose had been previously demonstrated to significantly reduce PTZ-induced C-SC seizure activity (Hewapathirane et al. 2008). For Ca^2+^ recordings, a higher dose of 15 mM PTZ was used after baseline recordings to ensure brains were still responsive, and to reduce latency to seizure time (Hewapathirane et al. 2008) so that tadpoles were not subjected to extended recording periods.

#### Pre-treatments with antiseizure drug valproic acid and the anti-inflammatory drug Losartan

Wild type tadpoles were arbitrarily divided into two groups. For each test, one group of 12 tadpoles was pretreated for two hours with either 5 mM Sodium valproate (VPA, Sigma) or 5 mM Losartan (potassium salt, Sigma), both dissolved in MQW, with the other untreated group receiving the same volume of MQW. VPA has been previously shown to reduce PTZ-induced seizures at 5 mM (Hewapathirane et al. 2008). Preliminary testing with 1, 2, and 5 mM doses showed that the 5 mM Losartan was effective in reducing PTZ-induced C-SC seizures. Some of the *NeuroD2* CRISPants, selected based on spontaneous seizure behavior, were pretreated with 5 mM Losartan prior to Ca^2+^ signal recordings and the BBB integrity assays. Untreated *NeuroD2* CRISPants were used as controls for comparison.

#### Losartan effect on C-SC seizure activity in NeuroD2 CRISPants

CRISPants were made using sgRNA rnk20 and raised to stage 47. Tadpoles were observed for 10 minutes and those displaying seizure-like C-SC activity were arbitrarily assigned to wells of a 24-well plate for video analysis (N = 18). Three consecutive 20 minutes recordings at 50 fps were made to determine baseline seizure activity, after which tadpoles were treated with 5 mM Losartan by adding the drug directly to the tadpole's medium in the wells. After two hours, three more recordings were taken, and all data analysed using TopScan to determine mean velocity in mm/sec for each tadpole before and after treatment. C-SC were hand counted frame by frame for each of the 18 tadpoles, and a before–after analysis was conducted to determine the effect of the drug on baseline seizure activity.

### Graphs and statistical analysis

All graphs and statistical analyses were prepared in GraphPad Prism v10. Raw data and analysis for all figures can be found in Supplementary File 1. The Shapiro–Wilk test for normality was used to confirm normal distributions, with nonparametric tests being used if distributions were nonnormal. Specific tests are included in figure legends.

## Results

### *Xenopus laevis NeuroD2* CRISPant tadpoles exhibit spontaneous seizures with an overlapping but distinct behavioral profile compared to PTZ-treated wild type tadpoles

*Xenopus* tropicalis *NeuroD2* CRISPant tadpoles were previously noted to have C-SC (Sega et al. 2019), similar to those elicited by exposure to epileptogenic drugs such as PTZ in wild type *X. laevis* tadpoles (Hewapathirane et al. 2008). *X. laevis* is allotetraploid, with around 60% of genes existing in both *L* and *S* chromosomal locations (Session et al. 2016). To first determine the usefulness of *X. laevis* as a genetic model for DEE, we designed sgRNA that would bind and target both copies of *NeuroD2*, to see if we could replicate the DEE72 phenotype (Fig. 1a). Editing was confirmed for all tadpoles and was associated with the expected reduction in NeuroD2 transcripts in the brain at stage 47 (Supplementary Fig. 1 in Supplementary File 2). We kept tadpoles in individual arenas so that they could not influence each other's behavior, and analysed video recordings to identify and quantify behavior.

Genotyping and other assays were performed after this step (Fig. 1b). Two sgRNA were designed to target the bHLH domain coding region of both *NeuroD2.S* and *NeuroD2.L*, “rnk5” resulted in a 15 bp deletion, removing 5 amino acids (aa) including part of the nuclear localization sequence (NLS). Despite the loss of 5 aa, the consensus NLS sequence KKRKMTK was recreated around the breakpoint (Fig. 1c). The second sgRNA, “rnk20”, targets the DNA binding region where the DEE72 causing variants are found in humans. This sgRNA causes frameshift InDels, most commonly a deletion of 4 bp, resulting in a truncated protein midway through the bHLH domain (Fig. 1c).

Automated tracking of movement from video recordings of individual tadpoles housed in 24-well plates showed increased activity in *NeuroD2* CRISPant tadpoles (Fig. 2a). Compared to wild type tadpoles induced to seizure behavior with PTZ, which swam consistently around the periphery of the arena, *NeuroD2* CRISPant tadpoles utilized the whole well. As a control for the effect of CRISPR reagent injection on behavior, we also tracked 12 tadpoles that had only been injected with the unloaded Cas9 protein. One of these Cas9 controls was very active, comparable to that of *NeuroD2* CRISPants, but the others appeared to behave similarly to uninjected control tadpoles, which either drifted slowly or swam for short periods around the periphery.

Previously, a type of induced seizure behavior, confirmed by both electrophysiology and Ca^2+^ signaling activity, has been described in *X. laevis* tadpoles as “C-shaped seizures”. Here, the trunk curls to one side in an extreme posture often resulting in the head touching the tail tip (Hewapathirane *et al*. 2008). Frame-by-frame analysis of video capture confirmed this behavior can be elicited by adding 5 mM PTZ to the tadpole's swimming water (Fig. 2b). While C-shaped postures were frequently seen in both PTZ-induced and *NeuroD2* CRISPant tadpoles, PTZ tadpoles were more likely to hold the posture for consecutive frames, indicating an acute convulsive state that resembles status epilepticus (Fig. 2b). PTZ-elicited seizures have been confirmed by both electrophysiological field potential recordings from the tadpole brain and Ca^2+^ signaling (Hewapathirane *et al*. 2008). Here, we refer to “C-SC” to describe the behavior seen in *NeuroD2* CRISPant tadpoles (Supplementary Video 1). Both individual frames with C-SC and C-SC clusters (where multiple C-SCs were detected, with an interval of less than 2 seconds between events), were manually counted from the same tadpoles for the 30 minutes of video-recorded footage (Fig. 2c, 2d). PTZ-induced tadpoles had the most C-SC (419 ± 40.2), compared to 335 ± 68.1 for rnk20 and 146 ± 44.6 for rnk5 *NeuroD2* CRISPant tadpoles (Fig. 2c, Supplementary Video 2). The truncating rnk20 *NeuroD2* CRISPant tadpoles therefore had an average of 2.3 times as many C-SC as the 5 aa deletion-causing rnk5. Kruskal–Wallis analysis of the C-SC data, with Dunn's post hoc testing of means, showed that rnk5 mean C-SC count was not increased over that of controls, while for rnk20, the higher mean C-SC count was not significantly different from PTZ-induced tadpoles. A similar relationship was seen with C-SC clusters (Fig. 2d), except that rnk5 was shown to have significantly more C-SC clusters than controls. Both sgRNA had significantly lower numbers of C-SC clusters compared with PTZ-treated tadpoles.

We also analysed the same videos with TopScan and found that mean velocity (mm/sec) was an excellent indicator of seizure activity, correlating strongly with C-SC counts (Fig. 2e; Supplementary Fig. 2 in Supplementary File 2). Conversely, TopScan automated scoring of C-SC was found to be inaccurate for *NeuroD2* CRISPant tadpoles (Supplementary Fig. 2a). On average, the truncating rnk20 *NeuroD2* CRISPant tadpoles swam 25 times faster than cas9 injected controls, and the 5aa deletion rnk5 *NeuroD2* CRISPant tadpoles swam 18 times faster than the controls. PTZ-treated tadpoles swam fastest with a mean velocity of 3.24 ± 0.23 mm/sec, compared to 2.27 ± 0.44 for Rnk20 and 1.58 ± 0.34 for rnk5 *NeuroD2* CRISPant tadpoles. Editing for both *NeuroD2* CRISPant groups was confirmed from Sanger sequence trace analysis using TIDE, with all tadpoles showing editing. For rnk20 tadpole editing varied from 3.5 to 33.3% with a mean of 15.6 ± 2.9%, and for rnk5 the range was 2.8–12.3, mean 6.4 ± 1.0 (Figure 2f). Because of the more obvious effect of the rnk20 edits on the NeuroD2 protein, as well as higher average editing, we proceeded with this sgRNA for further investigations.

### *NeuroD2* CRISPant tadpoles show strong, prolonged, and widespread Ca^2+^ signaling activity in the brain

**Fig. 3.**
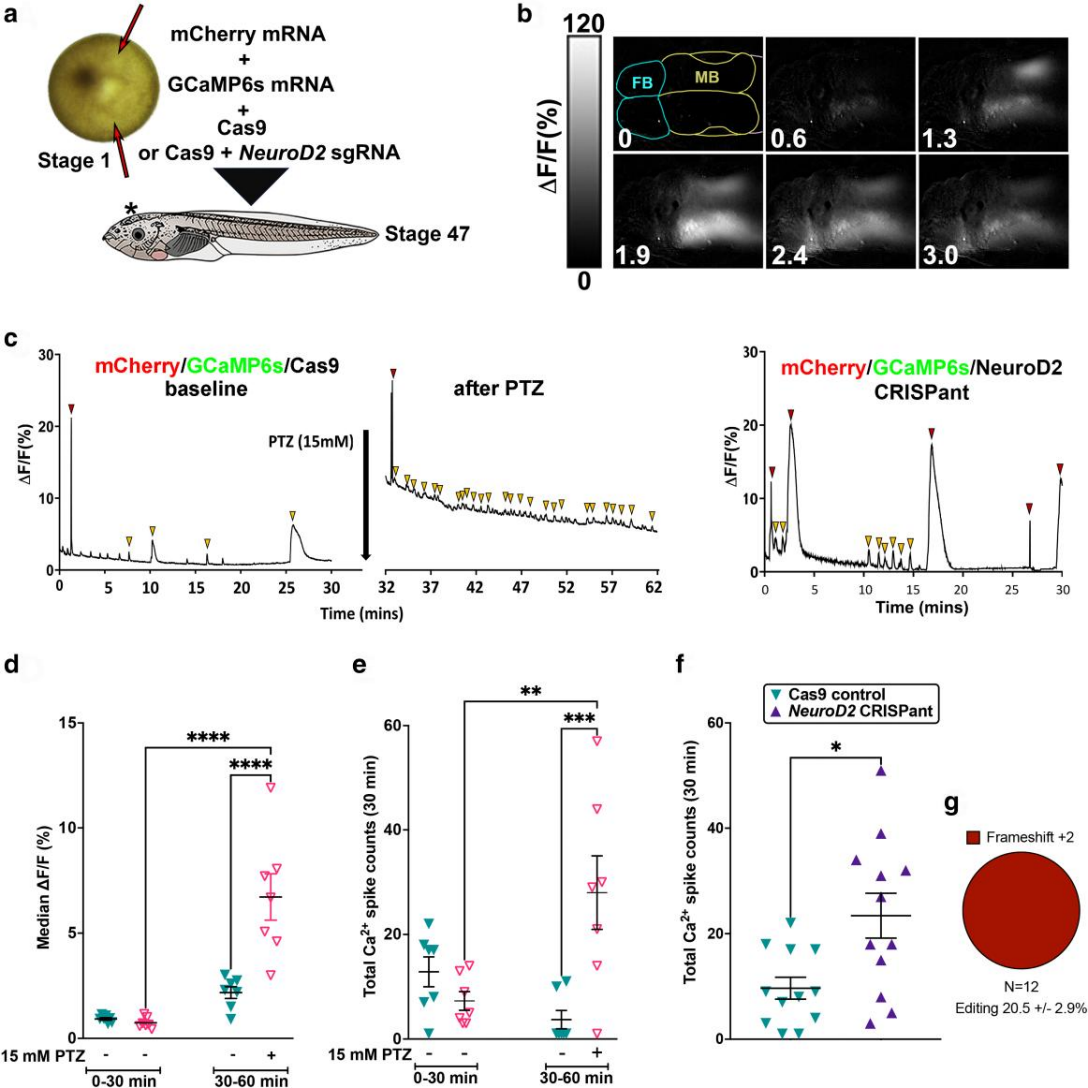
*NeuroD2* CRISPant tadpoles show strong, prolongued, and widespread Ca^2+^ signals in the brain. a) Experimental design. Fertilized *X. laevis* embryos at the one cell stage were injected twice (red arrows) on either side of the female nucleus (light spot), avoiding the ventrally located sperm entry point (dark spot). mCherry mRNA was used to confirm injection, GCaMP6s mRNA encodes a Ca^2+^-sensing fusion protein that can detect action potentials, emitting strong fluorescence in the GFP channel signals (Li *et al.* 2022). Controls were only injected with Cas9 protein, and CRISPant embryos were injected with Cas9 with *NeuroD2* sgRNA rnk20 loaded. Embryos were raised to stage 47. b) Example still frames taken every 0.64 minutes from a live *NeuroD2* CRISPant tadpole brain (TILLvisION 4.0). GCaMP6 s fluorescence (lighter colour = higher intensity) can be seen across large regions of the brain. Scale bar indicates Ca^2+^ signal intensity (%). Forebrain (FB) hemispheres outlined in blue, midbrain (MB) in yellow, hindbrain in pink is mostly out of shot. Time in minutes is indicated in the bottom left. c) Examples of Ca^2+^ signals in the midbrain optic tectum (MFI across 30 minutes), in control, PTZ-induced wild type tadpoles and *NeuroD2* CRISPants. Note that the PTZ-induced Ca^2+^ signals are relative to baseline activity (before PTZ application). Red arrowheads indicate large spikes and gold arrowheads small spikes. (d–f) Scatterplots showing individual tadpoles as triangles, horizontal bars are means and error bars show SEM. d) Median midbrain Ca^2 +^ signals in two groups of N = 7 Cas9 control tadpoles. One group was exposed to 15 mM PTZ to induce seizure activity in the second recording time window (30–60 minutes). 2-way ANOVA, post hoc test of all means (uncorrected Fisher's LSD) ****P < 0.0001 e) Comparison of total (large and small) Ca^2+^ spikes in the same tadpoles and groups as in panel d, 2-way ANOVA, **P < 0.01, ***P < 0.001. f) Comparison of total Ca^2+^ spikes in Cas9 control tadpoles vs *NeuroD2* CRISPants (N = 12) Mann–Whitney Test *P < 0.05). g) Summary of editing types detected by TIDE in the *NeuroD2* CRISPant tadpoles used to generate these data. Trace data for all tadpoles in the study can be found in Supplementary Fig. 3 in Supplementary File 2, raw data and statistics in Supplementary File 1.

To determine if the C-SC and faster swimming behaviors we detected in CRISPants were indicative of aberrant brain signaling, we used the genetically encoded Ca^2+^ indicator fusion protein GCaMP6s to record Ca^2+^ signals from tadpole brains at stage 47 (Fig. 3). Single-cell embryos were injected bilaterally with mCherry and GCaMP6s mRNAs in addition to the CRISPR reagents (Fig. 3a). Expression of mCherry in both halves of the neural plate at stage 16 was used to confirm delivery of reagents. At stage 47, tadpoles were first video recorded, and individuals with prominent C-SC were selected and mounted for live monitoring of Ca^2+^ signals over 30 minutes. Background recordings, or those from control, Cas9 only injected tadpoles (both N = 12), showed a generally low level of Ca^2+^ signals (observed as GFP fluorescence), but spikes of activity could still be detected (Supplementary Video 3). Some of these control tadpoles were then exposed to 15 mM PTZ and recording continued for a further 30 minutes. PTZ significantly increased Ca^2+^ signal intensity (median cross entire tadpole 6.7 ± 1.1%) compared to 0.7 ± 0.1% pre-PTZ exposure, or to 2.2 ± 0.3% in untreated siblings at the same timepoint (Fig. 3b; Supplementary Fig. 3 in Supplementary File 2, Supplementary Video 4). The average total number of large and small spikes was also significantly higher in PTZ-treated tadpoles than sibling controls (7.29 ± 1.78, Fig. 3e). *NeuroD2* sgRNA rnk20 CRISPants, on the other hand, showed additional strong, widespread signals that originated from a focal point before spreading across the entire brain on both hemispheres (Supplementary Video 5). These episodes, which generated large Ca^2+^ spikes, lasted between 3 and 5 minutes in some cases and may indicate epileptogenic activity (Fig. 3b and c; Supplementary Fig. 3 in Supplementary File 2). Even in between these episodes, Ca^2+^ signaling in CRISPant tadpoles was elevated, with increased numbers of smaller Ca^2+^ spikes. Total Ca^2+^ spike counts over 30 minutes was significantly higher in the *NeuroD2* CRISPant group (mean 23.42 ± 4.2, N = 12) than in Cas9 injected controls (9.67 ± 2.07, N = 12) (Fig. 3f). All CRISPant tadpoles were confirmed with editing in the range 7.7 to 42.9%, and 100% of edits resulted in frameshifts (Fig. 3g).

### *NeuroD2* CRISPant tadpole brains appear morphologically normal, but have a leaky BBB

We next looked to see if *NeuroD2* CRISPant tadpoles would develop gross brain morphological defects. We first utilized the unilateral mutant method pioneered by Willsey *et al.* (2021) in *X. tropicalis*, in a study of autism associated genes. CRISPR reagents (Cas9 loaded with *NeuroD2* rnk20 sgRNA) were injected into one blastomere at the two-cell stage of development, along with mRNA for nGFP. This results in one side of the embryo inheriting both the CRISPR reagents and nGFP, while the other side remains wildtype. At stage 19, the resulting embryos were sorted into left and right unilateral CRISPants, based on the GFP location. Embryos were raised to stage 47 and tadpoles photographed to compare the size and morphology of the left and right brain hemispheres. No differences in either were detected in 16 left or 19 right-sided unilateral tadpoles, obtained from two sibships (Supplementary Fig. 4 in Supplementary File 2).

**Fig. 4.**
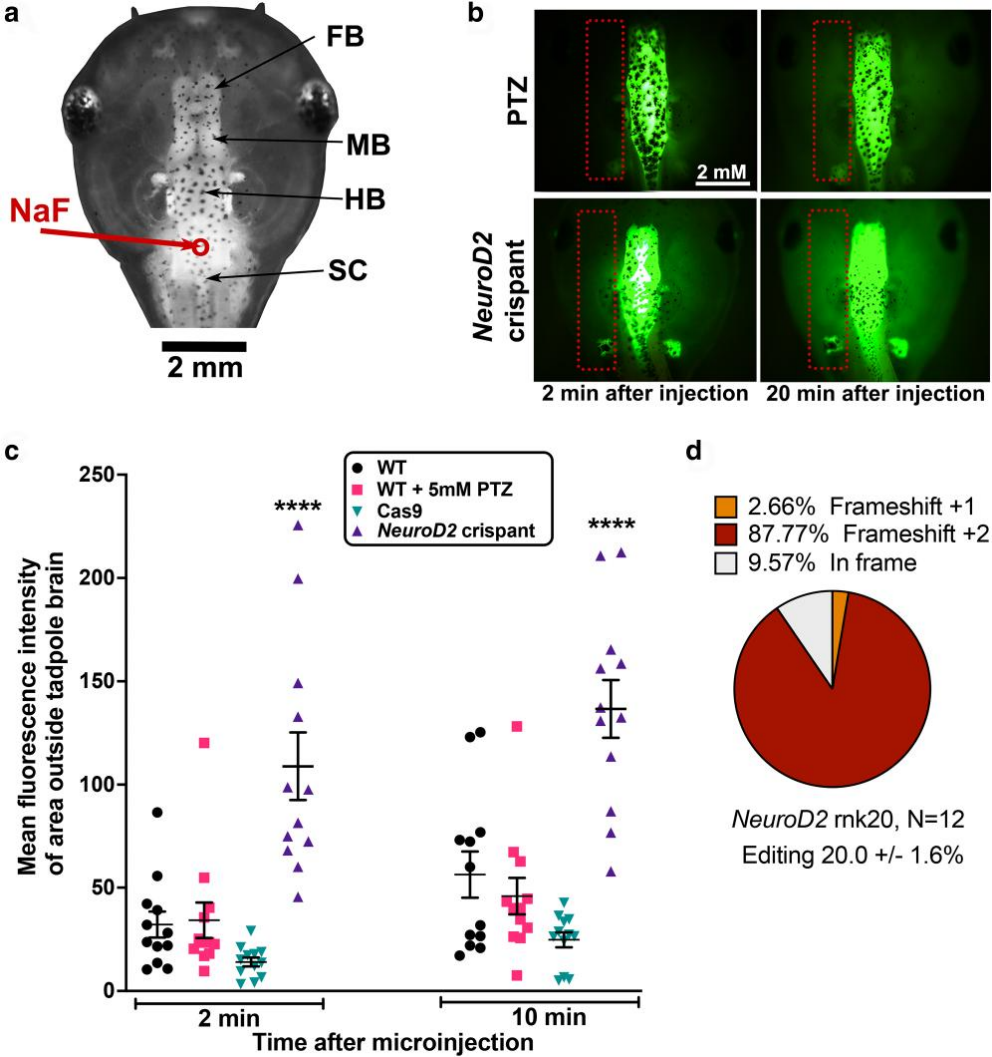
*NeuroD2* CRISPant tadpoles have a comparatively leaky BBB. a) Dorsal view of stage 47 tadpole head to show the parts of the brain and site of Na fluorescein (NaF) injection into the hindbrain ventricle (red circle and arrow). FB, forebrain; MB, midbrain; HB, hindbrain; SC, spinal cord. b) Examples of NaF dye injected tadpoles at 2 and 20 minutes after injection, visualized with GFP2 channel. Orientation of the tadpole head as in (a). Dotted rectangles show the area outside the brain that was used to calculate MFI. Scale bar in top left applies to all panels. Top, wild type (WT) tadpole treated with 5 mM of seizure-inducing drug PTZ for 2 hours prior to dye injection. Bottom, untreated *NeuroD2* CRISPant, sgRNA rnk20. c) Scatter plot showing MFI (dye leakage outside the brain) at 2 and 20 minutes post NaF dye injection for N = 12 tadpoles per group, 2-way ANOVA with Tukey post hoc analysis, ****P < 0.0001. Cas9 indicates tadpoles injected with Cas9 protein, but no sgRNA. d) Summary of tadpole editing in the *NeuroD2* CRISPant group, confirmed by Sanger sequencing and TIDE analysis. Raw data are in Supplementary File 1 and Supplementary Fig. 5 in Supplementary File 2.

We also measured the permeability of the BBB in both *NeuroD2* CRISPants and PTZ acute seizure induced tadpoles. NaF dye has been previously shown to be prevented from exciting the brain at stage 55 in uninjured tadpoles, indicating an intact BBB (De Jesus Andino *et al*. 2016). Adapting the method used in a recent study using *X. laevis* tadpoles to model traumatic brain injury (Spruiell Eldridge *et al*. 2022) for our younger tadpoles, we injected 9.2 nl of 0.1 mg/ml sodium fluorescein (NaF) into the 4th ventricle (Fig. 4a) at stage 47. BBB permeability was then measured by tracking dye distribution over 30 minutes (Fig. 4b). PTZ treatment to elicit acute seizures did not result in increased NaF loss from the brain compared to WT or Cas9 only injected controls at either timepoint. In contrast, *NeuroD2* CRISPants showed significantly higher NaF fluorescence outside the brain at both the 2 minutes (7.7 x higher) and 20 minutes timepoints (5.5 x higher), compared to Cas 9 controls (Fig. 4c). All CRISPant tadpoles were subsequently confirmed as edited (Fig. 4d). While all the groups showed some degree of NaF leakage after 20–30 minutes, the significantly higher and faster loss of NaF from brain of *NeuroD2* CRISPants indicates that the BBB is less robust in these tadpoles. Preliminary results from stage 47 brain transcriptomes combined with mined data from Ta *et al*. (2022) indicate that Vimentin (*Vim*), a marker of neural progenitors, decreases with age in developing tadpole brains and is higher in *NeuroD2* CRISPants brains than controls. Conversely, aquaporin1 (*aqp1*) which may play the role of aquaporin4, a mammalian BBB marker, in *Xenopus,* increased with age and was lower in CRISPants (Supplementary Fig. 6 in Supplementary File 2).

### The antiinflammatory drug losartan reduces both chemically induced and genetic seizure activity

ASD are front-line medications used in epilepsy to prevent seizures from occurring. However, there is a current lack of effective seizure controlling medication for treatment of DEE. Drug re-purposing for epilepsy, particularly the use of antiinflammatory and antioxidant medications with known, and manageable, side effect profiles, is increasingly desirable (for recent reviews, see Radu *et al*. 2017; Klein *et al*. 2020; Loscher 2020; Pawlik *et al*. 2021)). We tested two drugs, the commonly used antiseizure drug VPA, and the antiinflammatory drug Losartan, to determine the potential for seizure reduction. Losartan is a commonly prescribed drug used to lower blood pressure. It acts by inhibiting angiotensin II type I receptor antagonist, leading to up-regulation of the protease thrombospondin1 (TSP1). TSP1 can activate the proprotein form of the secreted paracrine factor TGFβ (Bar-Klein *et al*. 2014). Losartan has been previously reported to reduce the development of chronic seizures in rodent models of traumatic brain injury (Bar-Klein *et al*. 2014; Tchekalarova *et al*. 2016; Hong *et al*. 2019), but to our knowledge this drug has not been tested in models of genetic epilepsy such as DEE72.

**Fig. 5.**
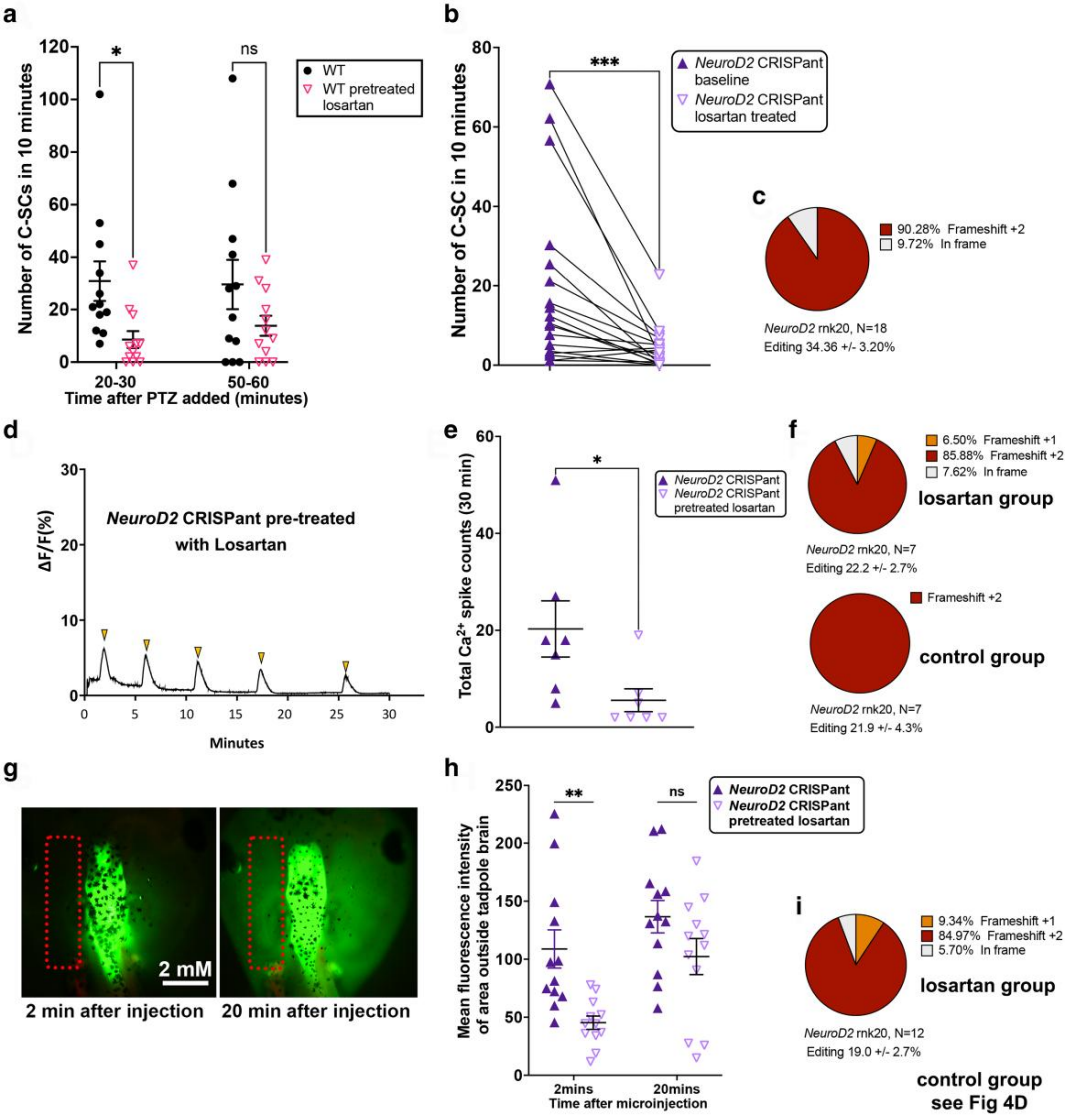
Pre-treating tadpoles with Losartan offers short-term protection from chemically induced or genetic seizure activity. a) Scatter plot of C-SC events in stage 47 wild type tadpoles recorded in 10 minutes either 20 minutes after induction of seizures with 5 mM PTZ or 50 minutes after. Pretreatment of tadpoles with 5 mM of the anti-inflammatory drug Losartan, compared to no pretreatment, N = 12 both groups. Analysis by 2-way ANOVA with Sidak's multiple comparisons test of means, *P < 0.05, ns = nonsignificant. b) Before–after plots of C-SC events for 18 *NeuroD2* CRISPant tadpoles, baseline events over 1 hour were counted. Two hours after addition of 5 mM Losartan to the tadpole medium, C-SC events were again counted for 1 hour. Wilcoxon rank test for matched pairs, triangles joined by black lines are the same tadpole, ***P < 0.001. c) Pie chart summarizing editing of the 18 *NeuroD2* CRISPants analysed in b. d) Example Ca^2+^ signal trace signaling in the midbrain optic tectum of a *NeuroD2* CRISPant tadpole pretreated with 5 mM losartan, with spikes of Ca^2+^ activity indicated by arrowheads. e) Scatterplot of total Ca^2+^ spikes, detected over 30 minutes, for two groups of N = 7 *NeuroD2* CRISPant tadpoles with or without losartan pretreatment. Mann–Whitney test, *P < 0.05. Trace files are in Supplementary Fig. 7 in Supplementary File 2. f) Summary of editing for each group of n = 7 tadpoles in panel e. All CRISPants were edited, and levels were not different between the groups (*P* = 0.96, unpaired t-test). g) *NeuroD2* CRISPant tadpole heads showing distribution of sodium fluorescein (NaF) dye 2 and 20 minutes after injection into the 4th ventricle. Dotted rectangle indicates the area outside the brain used to measure escaped NaF. h) Scatterplot comparing mean fluorescence intensity in the brain at 2 and 20 minutes post-NaF injection, for two groups of 12 *NeuroD2* CRISPant tadpoles, where one group was pretreated with 5 mM Losartan. Analysis by 2-way ANOVA with Sidak's multiple comparisons test of means, **P < 0.01, ns = nonsignificant. i) Pie chart summarizing NeuroD2 editing in the 12 tadpoles comprising the losartan group in panel h, controls are shown in Fig. 4d. All CRISPants were edited, and levels were not different between the groups (*P* = 0.76, unpaired t-test). Raw data can be found in Supplementary File 1 and Supplementary Figs. 5 and 8 in Supplementary File 2.

We first tested the effect of the drugs on PTZ-induced seizure behavior of wild type tadpoles. Tadpoles were pretreated for 30 minutes with 5 mM VPA, 5 mM Losartan or no drug, before adding 5 mM PTZ to the swimming water to induce seizures. The number of C-SC was counted in two 10-minute windows, 20–30 minutes and 50–60 minutes after adding PTZ (Fig. 5a, Supplementary 8a). At the earlier timepoint, the VPA pretreatment tadpole cohort had significantly fewer mean C-SCs than controls (Supplementary Fig. 8a, control mean C-SC: 103.2 ± 11.4, VPA mean C-SC 17.3 ± 3.8). The losartan pretreatment cohort also had significantly fewer C-SCs than controls, (Fig. 5a, control mean C-SC 30.8 ± 7.6, Losartan mean C-SC 8.5 ± 3.2). Despite their different models of action, both drugs were able to offer some protection from PTZ-induced seizure behavior, but the protection afforded by losartan did not extend to the later timepoint, suggesting it is short-lived in the acute seizure model. We also found that tadpoles in the VPA pretreatment group were significantly slower swimmers, whereas pretreatment with losartan had no effect on mean swimming velocity compared to controls (Supplementary Fig. 8b, c).

Since Losartan showed promise in the induced seizure model, we generated *NeuroD2* CRISPant tadpoles and raised them to stage 47. Because *NeuroD2* editing varies between tadpoles, the same animals were tested for a baseline seizure activity followed by a post treatment test. 18 tadpoles that were observed to have spontaneous seizure behavior were arrayed in 24-well plates and their activity was recorded for one hour at 50 frames per second. Frames with C-SC were counted by hand for each tadpole to get a baseline. Following 2 hours of treatment with 5 mM Losartan, the tadpoles were recorded for a further hour. C-SC were significantly reduced following Losartan treatment (baseline mean 19.66 ± 5.14 and post treatment mean 4.21 ± 1.27 C-SC per 10 minutes, *P* = 0.0002) (Fig. 5b). 16 of the 18 tadpoles had fewer C-SC after Losartan treatment, with an average of 4.7 times more C-SC seen in baseline recordings. All tadpoles were subsequently confirmed as edited (Fig. 5c). Velocity was also calculated using TopScan for all tadpoles. There was no difference in mean swimming velocities pre- and post-Losartan treatment (baseline mean 0.92 ± 0.22 and post treatment mean 0.56 ± 0.22 mm/sec, *P* = 0.32) (Supplementary Fig. 8d). Seven tadpoles swam faster on average, nine swam slower, and two were unchanged after treatment.

We next investigated whether Losartan pretreatment could reduce the aberrant brain signaling activity seen in our *NeuroD2* CRISPants. We generated CRISPant tadpoles with mRNA encoded GCaMP6 s and arbitrarily assigned them to a Losartan pretreatment or control groups of N = 7, to observe the effect GCaMP6 s fluorescence (indicating Ca^2+^ signaling) in the stage 47 brain (Fig. 5d, e). Significantly fewer Ca^2+^ spikes were seen in the group of *NeuroD2* CRISPant tadpoles treated with 5 mM Losartan for 2 hours prior to recording, nearly a 4-fold reduction. Untreated CRISPant tadpoles had a mean of 20.3 ± 5.8 spikes in 30 minutes compared to the Losartan pretreated batch with 5.6 ± 2.4 spikes in 30 minutes. Editing was confirmed for all tadpoles, with no difference between treatment groups (N = 7 per group, Fig. 5f). We also examined the effect of 2 hours of pretreatment with 5 mM Losartan on the BBB integrity of *NeuroD2* CRISPants using the NaF assay (Fig. 5g), comparing to controls from Fig. 4c. *NeuroD2* CRISPant tadpoles in the Losartan pretreatment group were initially better able to retain the NaF dye in the brain (MFI outside the brain was 108.80 ± 16.43 for controls and 45.32 ± 5.76 for the Losartan group). However, analysis at 20 minutes after injection showed there was no difference between the two groups (mean 136.6 ±/ 13.98 for controls and 102.3 ± 15.53 for Losartan) (Fig. 5g). Editing was again confirmed for all tadpoles, with no difference between treatment groups (N = 12 per group, Fig. 5i and 4d).

## Discussion

### Tadpole behavior analysis can be useful phenotyping tool for preclinical models of DEE

Tadpoles of *X. laevis* frogs develop along a well-defined series of morphologically described stages (Nieuwkoop and Faber 1967). Since developmental rate is dependent on temperature for this species, we chose to evaluate behavior at stage 47. If feeding is not initiated at this stage, tadpoles remain in developmental stasis, allowing time for behavioral observations. We observed a considerable amount of batch variability in baseline behavior in this study, with Cas9 or uninjected control tadpoles barely moving at all (Fig. 1) compared to velocities of 4.8 mm/sec in Fig. 4. Currie *et al.* (2016) looked at the development of swimming behavior in *X. laevis* tadpoles. They measured swimming velocity in a 50 mm arena and showed that tadpoles began swimming at stage 45, but by stage 47 they were active almost continuously and swam at a mean velocity of 10 mm/sec. While we used a smaller arena size (17 mm diameter) this is unlikely to account for the magnitude of these differences. It is possible that our practice of withholding food to induce developmental stasis contributes, and further work will be needed to ascertain if this is the case. Alternatively, our tadpoles could be swimming less due to exposure to strong light, which is used for video recording. *X. laevis* tadpoles sense light via the pineal gland (Jamieson and Roberts 2000).

Automation of behavioral characteristics using TopScan in 24-well plate arrayed tadpoles has been previously described (Panthi *et al*. 2023). In *NeuroD2* CRISPants, mean swimming velocity was elevated compared to controls, and is a good indicator of hyperactivity, also described in mouse *NeuroD2* knockouts (Runge *et al*. 2021). Swimming track data highlighted differences in the occupancy of the arena, with *NeuroD2* CRISPants tending to cross the center more frequently than PTZ-induced acute seizure tadpoles. This also seems to mirror the mouse knockout (Runge *et al*. 2021). While others have reported a recapitulation of the PTZ-induced behavioral phenotype in *NeuroD2* CRISPants of the diploid *X. tropicalis*, in isolated *X. laevis* tadpoles we were able to detect differences in the two behaviors, with CRISPants more often just showing C-SC in single frames and having more periods of inactivity between C-SC clusters. For PTZ-induced tadpoles, TopScan was able to identify C-SC accurately, using elongation ratio thresholds (altered length/width), as confirmed by manual counts. On the other hand, spontaneous C-SC of the CRISPants were not reliably detected, and needed to be manually scored on a frame-by-frame basis, possibly due to the more transient nature of the C-shaped postures. We note this difference from Sega *et al.*’s (2019) model of DEE72 in *X. tropicalis,* where much stronger seizure activity was reported with tadpoles spending up to half of their time in C-SC at stage 47, when multiple tadpoles were in the same dish. This difference could be due to our tadpoles being individually housed, with no opportunity to influence each other's behavior.

### Both truncating edits and the deletion of amino acids in the DNA binding domain result in seizure-like behavior

We used two sgRNA with different predicted outcomes in this study. For sgRNA rnk20, we obtained sequencing data for 24 stage 14 embryos and 73 stage 47 tadpoles after phenotyping (Supplementary Fig. 1a). InDelphi predicts a predominant 4 bp deletion, which was confirmed from TIDE analysis of Sanger sequence traces in all 73 sequenced tadpoles, as well as in 22 of 24 embryos. The second highest prediction by InDelphi for this target is a 13 bp deletion, which was detected in 7/24 embryos (29%) and 27/73 tadpoles (37%). We conclude that the most common outcome for this sgRNA is a deletion of 4 bp which results in a change of amino acid residue at position 135, followed by a stop codon (nonsense mutation). The outcome of the less common 13 bp deletion is the same, as both deletions result in +2 frameshift. Any protein product formed from rnk20 sgRNA editing is therefore likely to be truncated just 3′ of the NLS, with a shortened bHLH DNA binding domain (Fig. 1c). We expect this to represent a mosaic loss of function phenotype. In mice, loss of *NeuroD2* results in ataxia and death by postnatal day 14 (Olson *et al*. 2001). For the rnk5 sgRNA, InDelphi predicts a 15 bp deletion, which was observed in half of our tadpoles (5/10, Supplementary Fig. 1b). This creates a deletion of 5 amino acids in the region of the NLS, but the NLS itself is preserved (Fig. 1c). The outcome of this *NeuroD2*Δ5 deletion is less clear. Perhaps surprisingly, this sgRNA was also able to induce C-SC behavior, even with relatively low editing levels. Potentially, the loss of 5 amino acids in the bHLH domain may affect the structure of the transcription factor, resulting in reduced function. Our results suggest that embryos are very sensitive to *NeuroD2* protein levels during early development. Sega *et al.* (2019) found that overexpression of wild type *NeuroD2* led to the production of ectopic primary neurons, whereas the DEE72 variant replicating *NeuroD2* alleles were either less active or not active. This suggests that DEE72 is caused by loss of function of *NeuroD2*, and patients are haploinsufficient. Haploinsufficiency is also supported by the mouse model (Runge *et al.* 2021). Here, we show that even a relatively small decrease in functional *NeuroD2* results in a detectable C-SC phenotype. The two original DEE72 variants, Glu130Gln and Met134Tyr, are predicted to alter DNA binding (Sega *et al.* 2019), and both have been linked to DEE in at least one other patient (Runge *et al.* 2021; Demarest *et al.* 2022; Sakpichaisakul *et al.* 2022). However a third variant in the DNA binding domain, Arg129Trp, was identified in a patient with neurodevelopmental delay but no epilepsy (Runge *et al.* 2021). Two further variants, Leu163Pro (inside bHLH domain) and His268Gln have also been associated with neurodevelopmental phenotypes/autism, but not with seizures (Mis *et al.* 2021; Runge *et al.* 2021).

### C-shaped contractions resemble the Mauthner neuron-mediated C-start responses of larval fish and amphibians

Aquatic vertebrates such as fish and larval frogs (tadpoles) have a unique pair of giant axon neurons called Mauthner neurons. In zebrafish, one Mauthner neuron on each side of the animal projects its axon from the 4th rhombomere of the hindbrain to the spinal motor neurons on the contralateral side (Kimmel *et al.* 1974). The giant axons make the Mauthner neurons capable of rapid responses. Typically, the input to the Mauthner neurons is either from the nearby sensory auditory afferent neurons or from the vibration sensing lateral line cells (Sillar 2009). In *Xenopus* tadpoles, Mauthner neurons have been associated with a stereotypical fast escape response termed the C-start (Sillar and Robertson 2009). In teleost fish, Mauthner neurons are well established as the command neurons for the C-start escape response (Sillar 2009). The C-shaped seizures described by (Hewapathirane *et al.* 2008; Sillar and Robertson 2009; Sega *et al.* 2019) may reflect inappropriate firing of the Mauthner neurons, resulting in a posture that mimics that of the C-start. In support of this, Zarei *et al* (2017) found that removal of one otic vesicle (ear) resulted in all the C-start bends going in the same direction instead of 50:50 left or right. We found that our C-SC were of longer duration than the C-starts induced by temperature stress (Sillar and Robertson 2009), in stage 42 tadpoles, which last about 40 msec in total, or stimulated C-starts in stage 46 tadpoles, which lasted around 30 msec (Zarei *et al.* 2017). PTZ-induced C-SC were seen to last for about 160 msec, and the spontaneous C-SC of *NeuroD2* CRISPants 40–80 msec. In future, it may be possible to target GCaMP to Mauthner cells to see if these neurons are indeed activated in C-SC.

### The tadpole model reveals that disrupted brain activity and a leakier BBB results from haploinsufficiency of *NeuroD2*

Tadpole models of induced acute seizures had been previously described (Hewapathirane *et al.* 2008; Bell *et al.* 2011). The use of tadpole CRISPants to confirm a novel DEE resulting from de novo human variants that result in loss of function of one copy of *NeuroD2* (Sega *et al.* 2019) additionally demonstrated the utility of this model in functional studies of genetic epilepsy. Here, we have demonstrated that the allotetraploid *X. laevis*, which is better established as a neuroscience model, can also be used for genetic epilepsy. Ta *et al*. (2022) recently provided a comprehensive transcriptome of brain development in *X. laevis*, interrogating these data show that only the *NeuroD2*.S homeolog is expressed in the brain. Expression is mostly in the forebrain and midbrain, and midbrain expression was seen to increase steadily from stage 44 to stage 61 (Ta *et al.* 2022).

Sega *et al.* showed that C-SC, resembling those seen in PTZ-induced epilepsy, can be observed from stage 43, increasing in frequency until stage 47. Here, we have focused on stage 47 tadpoles as this provides a natural stalling point in tadpole development in the absence of feeding, allowing time for multiple analyses. Knockout of *NeuroD2* in a mouse model was previously shown to result in microcephaly, ataxia, and death at approximately 2 weeks (Olson *et al.* 2001). In a more recent examination of *NeuroD2* knockout mice focusing on the cerebral cortex, Runge *et al.* (2021) showed altered corticogenesis, excitatory synapse density and turnover, resulting in hyperexcitable layer five neurons. Social interactions, stereotypical behaviors, hyperactivity, and occasional seizures were noted in both homo- and heterozygote KO mice. Both behavioral aspects and seizures were recapitulated in conditional knockouts of *NeuroD2* in forebrain excitatory neurons, but *NeuroD2* has a much broader expression in both the human brain (Human Protein Atlas) and the tadpole brain (Ta *et al.* 2022). We observed seizures in all edited tadpoles, which may be more obvious due to continuous monitoring of the isolated animals. Ca^2+^ signaling observations showed widespread neuronal hyperactivity, confirming that C-SC and hyperactivity are accompanied by an underlying aberrant neuronal signaling. *NeuroD2* was also found to be significantly decreased at both transcript and protein level in a model of Zika virus induced microcephaly (Fujimura *et al.* 2023). We did not observe any effect on gross brain morphology in the tadpole model, since unilateral CRISPants had symmetrical brains. The integrity of the BBB is linked to the development of seizures in acquired epilepsy (reviewed in (Gorter *et al.* 2015)), but has not to our knowledge been linked to genetic DEE. Our work suggests that the BBB may be less effective at stage 47. The BBB has not been investigated in NeuroD2 pathology before, but in one report of a child with *NeuroD2* M134T variant, and one with E130Q, the corpus callosum was noted to be thin in MRI reports (Sega *et al.* 2019; Demarest *et al.* 2022). Interestingly, PTZ treatment over the same timeframe had no effect on BBB integrity, so this aspect is most likely neurodevelopmental rather than result of seizure activity in the brain.

### The antihypertensive drug Losartan shows promise for re-purposing as a seizure control drug in DEE72

In the initial report of DEE72, neither child's seizures could be controlled by treatment with ACTH, prednisolone, or vigabatrin and a number of other ASD (Sega *et al.* 2019). Seizure freedom was reported for the patient with the more severe E130Q variant by using the ketogenic diet, and for the M134T patient by means of vagal nerve stimulation (Sega *et al.* 2019). This highlights the need to explore alternative mechanisms of treating DEE. Angiotensin II receptor transcripts were found to be significantly elevated in a rat model of epilepsy (Pereira *et al.* 2010). These authors first demonstrated the anticonvulsive effect of Losartan, an angiotensin II receptor antagonist antihypertensive drug, in their model. Losartan may protect from epileptogenesis by preventing TGF-β signaling in astrocytes (Bar-Klein *et al.* 2014). Here we have shown that pretreatment of CRISPant tadpoles with the losartan both improves the integrity of the BBB and reduces the number of Ca^2+^ spikes in our tadpole model. Further, 17 out of 18 NeuroD2 CRISPants treated with Losartan had fewer C-SC events compared to baseline, amounting to a 4-fold reduction on average. In contrast, tadpole mean velocity was not significantly affected, suggesting tadpoles” overall activity is not impaired by Losartan. Previously, Losartan has been shown to attenuate BBB permeability in the lithium-pilocarpine model of epilepsy in rats (Hong *et al.* 2019). It is one of several non-ASD that have been shown to have potential in the treatment of acquired epilepsy in humans (reviewed in Du *et al.* (2016)). A recent retrospective study showed significantly reduced epilepsy incidence in a cohort of arterial hypertension patients from Germany who were being treated with six different angiotensin II receptor blockers (Losartan, Valsartan, Telmisartan, Olmesartan, Candesartan, Irbesartan). The same study showed that Losartan alone associated with a significantly lower incidence of epilepsy (Doege *et al.* 2022). Losartan was also effective at reducing seizures and associated neuronal damage in a mouse model of hypertension and epilepsy (Tchekalarova *et al.* 2016). Conversely, Reyes-Garcia *et al.* (2019) reported that Losartan did not reduce epileptiform activity in resected brain slices from human patients of drug-resistant epilepsy. Since the effect on the BBB integrity in the tadpole model was eventually overcome, Losartan pharmacokinetics may play a role in this variability.

### Conclusions and limitations of the study

We have increased the toolkit for assessing seizure activity in *Xenopus* tadpole models of DEE, by using automated detection of mean tadpole velocity and live imaging of hyperactive brain activity using Ca^2+^ imaging. We have also identified a possible failure of BBB integrity or development in DEE72, and have identified Losartan as being a potentially useful drug for reducing seizures. As DEE is genetically diverse, it remains to be seen whether BBB integrity is a common associated feature, or whether Losartan may be generally useful in preventing epileptogenesis in DEE. Even though our colony has been closed for nearly 20 years, tadpoles come from a genetically diverse population of *X. laevis* and not from an isogenic line. Taken alongside CRISPR/Cas9 variation in editing and the likelihood of mosaicism, this means that even with sibling controls, we do note a considerable variation in tadpole outcomes. Since CRISPants are mosaic, our editing information for tadpoles may not perfectly reflect the situation in the brain. In future, it may be more informative to assess editing in extracted brains, rather than whole tadpoles. Scoring of C-SCs had to be done manually and could not be reliably automated with TopScan. A mechanism for detecting and tallying these rapid C-start like movements would be advantageous in developing these models for use in preclinical drug screening.

## Data Availability

All data presented are in the manuscript or in the Supplementary data (Supplementary Figs. 1-8 and Supplementary Data File 1). Supplementary Videos 1-5 and Supplementary Files 1-2 are linked to figshare: https://doi.org/10.25386/genetics.25649046.
