# Correction to: Knockdown of *NeuroD2* leads to seizure-like behavior, brain neuronal hyperactivity and a leaky blood-brain barrier in a *Xenopus laevis* tadpole model of DEE72

**DOI:** 10.1093/genetics/iyaf010

**Published:** 2025-02-04

This is a correction to: Sulagna Banerjee, Paul Szyszka, Caroline W Beck, Knockdown of *NeuroD2* leads to seizure-like behavior, brain neuronal hyperactivity and a leaky blood-brain barrier in a *Xenopus laevis* tadpole model of DEE72, *Genetics*, Volume 227, Issue 3, July 2024, https://doi.org/10.1093/genetics/iyae085

In the title and throughout the body of the article, erroneous references to ‘DEE75’ have been corrected to read ‘DEE72’.

